# Bilateral Renal Replacement Lipomatosis: A Case Report on Rare Complication of Obstructive Uropathy

**DOI:** 10.7759/cureus.16596

**Published:** 2021-07-23

**Authors:** Gautam Jesrani, Samiksha Gupta, Tagru Raju, Nidhi Bhardwaj, Monica Gupta

**Affiliations:** 1 General Medicine, Government Medical College and Hospital, Chandigarh, Chandigarh, IND

**Keywords:** renal replacement lipomatosis, obstructive uropathy, chronic renal failure, urinary tract infection, xanthogranulomatous pyelonephritis

## Abstract

Renal replacement lipomatosis (RRL) is an uncommon complication, leading to the fatty replacement of the renal parenchyma. Various etiologies have been described for this long-term infirmity, but obstructive uropathy is one paramount cause. Previously described reports have documented unilateral disease in the majority, but we are narrating a case of bilateral RRL, which is very scarce in the literature. A 57-year-old man, who was a known case of obstructive uropathy, presented to us with the symptoms of urinary tract infection. In imaging evaluation, the patient was found to have bilateral RRL.

## Introduction

Renal replacement lipomatosis (RRL) is a chronic degenerative process, in which renal parenchyma is replaced by fatty tissue, and long-term obstructive uropathy is the most common identified etiology [1]. It is a rare complication and mostly described literature is for unilateral disease. According to one research, the incidence of RRL was found to be 0.66% in 3,500 cases of intravenous pyelogram and the ailment has no sex preponderance [2]. In the past, histopathological examination was pivotal for the diagnosis, but immense use of imaging studies has improved our knowledge to diagnose the disease in a non-invasive manner.

## Case presentation

A 57-year-old gentleman presented to us with complaints of burning micturition from the last 10 days and on and off fever from the last six days. He has no history of hematuria, abdominal pain or decreased urine output in the current illness. The patient was a known case of obstructive uropathy and chronic renal failure diagnosed two years back. At that time, he had similar complaints of the urinary system and was diagnosed with bilateral ureteric calculi and hydronephrosis. For this, D-J stents were inserted on both sides, along with antibiotics for urinary tract infection. He recovered in the next three months and both of the stents were removed in subsequent one-month duration but suffered from chronic stable renal insufficiency afterward (baseline creatinine of 2.0-2.5 mg/dL).

On presentation, the patient had leukocytosis (18,000 per microliter), urea of 105 mg/dL (normal <45) and creatinine of 3.2 mg/dL (normal 0.8-1.8). Urine analysis depicted 25-30 pus cells per microliter of urine, along with mild albuminuria and culture had growth of *Escherichia coli* (>10^5^ colony-forming unit). The patient was managed with intravenous antibiotics (Piperacillin Tazobactam 4.5 g twice a day), according to the culture sensitivity and renal dose modification. Imaging studies were instituted as the patient was having obstructive uropathy in the past. His ultrasound abdomen demonstrated bilaterally enlarged kidneys with multiple calculi in both of the renal pelvis and probability of hydronephrosis.

**Figure 1:**
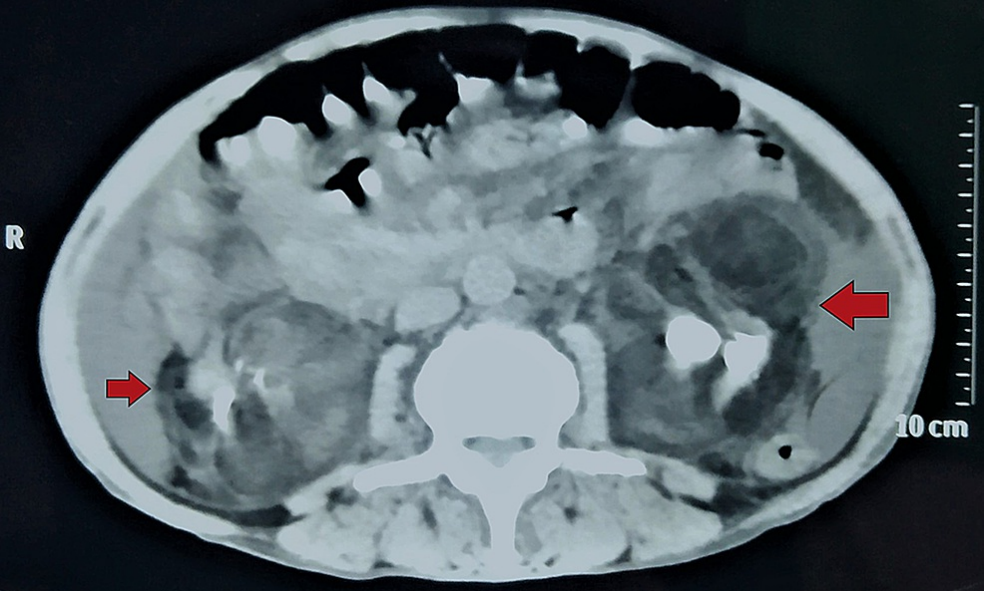
Non-contrast computed tomographic scan of the abdomen (axial section) demonstrating bilateral renal parenchymal replacement with fatty tissue (red arrow) and calculi in the pelvis.

**Figure 2:**
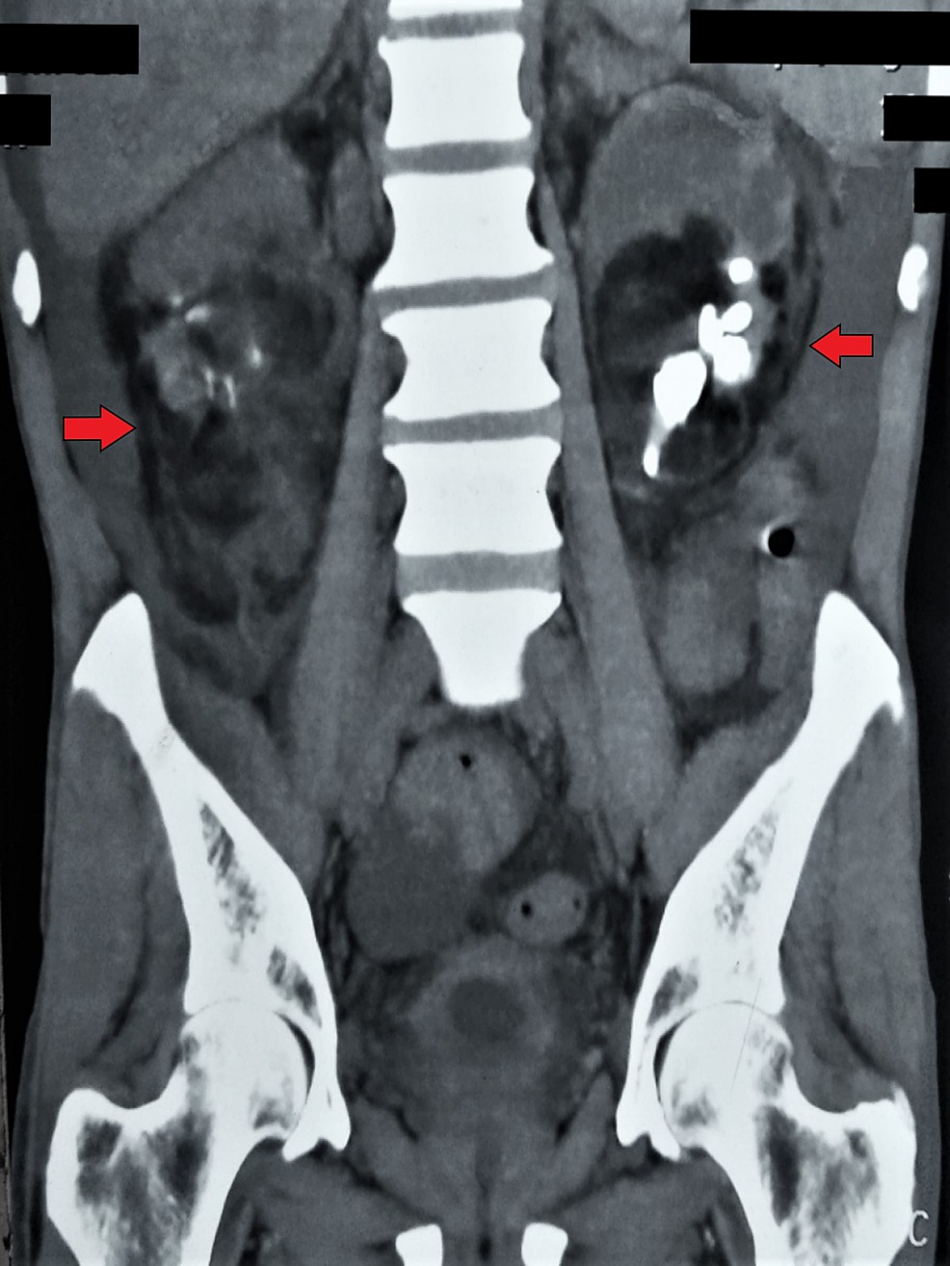
Reconstructed coronal section of the non-contrast computed tomographic scan of abdomen demonstrating bilateral renal replacement lipomatosis (red arrow) and multiple renal calculi.

On further evaluation, the patient underwent non-contrast computed tomography (CT) of the abdomen, which described the replacement of both renal parenchyma and perinephric space by fat attenuation density (-40 to -60 Hounsfield units, HU) with multiple calculi, causing dilatation of pelvi-calyceal system bilaterally (Figures 1, 2). Also, there was atrophy of the remaining renal parenchyma at the upper poles bilaterally. These findings were suggestive of RRL as the adipose tissue replacement was seen in the perinephric space and renal sinuses, without any evidence of inflammation as seen in xanthogranulomatous pyelonephritis (XGPN). Then, the patient underwent a renal diethylene triamine penta-acetic acid (DTPA) scan, which demonstrated a split renal function of 33% on right and 39% on the left side. His symptoms improved with initial conservative management and serum creatinine reached the pre-disease level (2.4 mg/dL). The patient was advised for tissue biopsy diagnosis and surgical removal of the involved part, but refused any invasive procedure, after understanding the possible outcomes. He was discharged on appropriate antibiotics after seven days of hospital stay and advised for close follow-up with the urology department.

## Discussion

RRL is a rare benign pathology of the kidney, leading to renal parenchymal atrophy. Chronic inflammatory induction of fatty tissue emergence and compensatory fatty replacement of atrophied renal parenchyma are two suggested pathologies [3]. Renal sinus lipomatosis is one, mild variety of RRL, in which there is a fat replacement from peripelvis to renal sinuses only and rarely associated with any symptoms. In severe forms of RRL, the entire renal parenchyma is replaced with adipose tissue and long-standing obstruction, inflammation and infections are the predominant elements in this process [4,5]. There are no disease-specific symptoms and commonly the patient presents with dull flank pain, fullness or urinary tract infection.

Histopathological examination was foremost in the past for the diagnosis of RRL, but CT and magnetic resonance imaging techniques have formulated this process undemanding. XGPN and angiomyolipoma are two close differentials for RRL, which can also be ruled out by CT scan features. CT findings of XPGN include reniform mass (-15 to 29 HU), calculi with the peripheral enhancement of compressed residual parenchyma, collectively giving an appearance of “grape bunch” or “claw of bear” [3]. Likewise, in RRL, imaging demonstrates the presence of fat tissue intensity inside the renal sinuses and perirenal space with HU of -20 or lower, which is confirmatory for adipose tissue [3]. Additionally, to distinguish RRL from other important renal involving diseases, we have included various radiological and histological features of XPGN, renal angiomyolipoma and liposarcoma in Table 1 [3,6-11].

**Table 1:** Clinical, radiological and histo-pathological features of close differentials of renal replacement lipomatosis. * Hounsfield unit

Disorder	Clinical features	Radiological features	Histo-pathological features
Renal replacement lipomatosis	No symptoms in mild variety; flank mass, swelling, pain, fever, hematuria and weight loss.	Negative attenuation fatty tissue (-20 HU* or less) inside the renal sinus, perinephric space or complete renal parenchyma replacement.	Bright yellow fat on gross examination and microscopically, lipid laden macrophages outside the true renal parenchyma with clear demarcation and atrophy of residual renal tissue.
Renal angiomyolipoma	Flank pain, hematuria, hemorrhage, palpable mass, anemia and urinary tract infection.	Mass of non-homogenous intensity (tissue attenuation according to a predominant component, i.e., fat, blood vessel or muscle); hemorrhage, and less commonly, necrosis inside the mass (fluid density).	Varying fractions of atypical blood vessels, fatty tissue and smooth muscle. Epitheloid angiomyolipima includes a polygonal cell structure with eosinophilic cytoplasm and aberrant nuclear structure.
Xanthogranulomatous pyelonephritis	Pain, fever, palpable mass, gross hematuria and weight loss.	Multiple areas of low attenuation (-15 to 30 HU*) with peripheral enhancement (active inflammation in residual renal tissue) and calyceal dilation; collectively demonstrating an appearance of “grape bunch” or “claw of bear”.	Pale yellow fat on gross examination and microscopically, lipid-rich macrophages inside the true renal parenchyma and no clear demarcation of fat invasion.
Renal liposarcoma	Flank pain, palpable mass, loss of appetite, weight reduction, hypertension and rarely hematuria.	Contrast-enhanced heterogeneous mass, encroaching retroperitoneal structures with varying proportions of fatty tissue, and presence of septa.	Three variants: Well-differentiated, Myxoid and Pleomorphic. Pathognomonic lipoblasts can be seen microscopically (identified by round lipid vacuole in cytoplasm, chromatin spikes, sometimes signet ring appearance and cell size less than mature adipocyte).

Currently, there is no identified treatment for this complication, but early removal of the triggering factor can halt the progression [12]. In complete mutilation of renal parenchyma, RRL can manifest as end-stage renal disease and is resulted due to a prolonged process. A relatively less severe malady may have some preserved renal functions, like in our case, which should be assessed before planning any surgical intervention. For residual renal function valuation, intravenous urography can be used, but DTPA scan is more superior in this context. Further, the presence of large RRL with inappreciable renal function warrants complete nephrectomy, whereas small lesions can be managed with renal sparing interventions. Following surgical removal, histopathological evaluation is advisable for better exclusion of other close differentials and concealed malignancy.

## Conclusions

To conclude, RRL is a rare consequence of common obstructive uropathy and can lead to permanent loss of renal functions. Imaging studies are crucial in diagnosing this entity and should be promptly utilized in suspected individuals. For this, USG of the renal system is a valuable screening modality and a CT scan should be employed in the high index of suspicion. Being an irreversible pathology, surgical removal is most commonly offered treatment, but early mitigation of the offending source can halt the progression. Therefore, timely recognition of risk factors is paramount for a favorable outcome.
